# Near-infrared spectroscopy combined with machine learning for plasma-based discriminant diagnosis of malignant mesothelioma: a retrospective study

**DOI:** 10.7717/peerj.20503

**Published:** 2025-12-19

**Authors:** Yixuan Gu, Ruting Wang, Weimin Mao, Zhongjian Chen

**Affiliations:** 1 Department of Medical Oncology, The Second School of Clinical Medicine of Zhejiang Chinese Medical University, Hangzhou, Zhejiang, China; 2 Zhejiang Cancer Hospital, Hangzhou, Zhejiang, China; 3 Zhejiang Key Laboratory of Diagnosis & Treatment Technology on Thoracic Oncology (Lung and Esophagus), Hangzhou, Zhejiang, China

**Keywords:** Malignant mesothelioma, Discriminant diagnosis, Machine learning, Near-infrared spectroscopy, Clinical application

## Abstract

This study developed a novel, non-invasive platform integrating near-infrared spectroscopy (NIRS) with machine learning (ML) to address the critical clinical challenge of misdiagnosing malignant mesothelioma (MM). We analyzed plasma samples from 99 individuals (29 MM, 41 lung cancer (LC), and 29 healthy controls). A support vector machine (*SVM*) model perfectly discriminated MM from LC (area under the curve (AUC) = 0.827), while a partial least squares (*PLS*) model differentiated MM from healthy control (HC) with high accuracy (AUC = 1.0). Despite the highly promising results, this single-center study is however limited by a small sample size, inherent to the rarity of MM and the associated difficulties in patient recruitment. Our findings demonstrate the potential of the NIRS-ML platform as a highly accurate tool for improving MM diagnosis and discriminant diagnosis, meriting further validation in larger cohorts.

## Introduction

In China, malignant mesothelioma (MM) is a rare tumor associated with asbestos exposure or genetic factors (Zhao et al., 2017). Presently, the diagnosis of MM poses numerous challenges. Firstly, although biomarkers such as mesothelin and HMGB1 are in clinical use (Chen et al., 2017), no highly specific diagnostic marker for MM has been established, and research in this area remains ongoing. Secondly, owing to the rarity of MM and limited clinical exposure, it is frequently overlooked during discriminant diagnosis. Thirdly, nonspecific imaging features further complicate accurate identification by radiologists. Therefore, improving early detection and diagnosis of MM is urgently needed.

The discriminant diagnosis of MM remains challenging due to its non-specific clinical presentation, which includes chest pain, dyspnea, fatigue, and persistent cough, symptoms that often overlap with those of lung cancer (Addis & Roche, 2009; Cavone et al., 2019; Guo et al., 2017). MM is frequently asymptomatic, leading to delayed medical consultation and advanced-stage detection. Misdiagnosis as lung cancer may result in inappropriate treatment and disease progression (Ostroff et al., 2012). Thus, there is an urgent need for reliable discriminative diagnostic methods to distinguish MM from confounding diseases and facilitate early screening in high-risk populations. This is particularly critical within China’s tiered healthcare system, where primary institutions serve as the first point of contact and play an essential role in initial diagnosis and triage.

Near-infrared spectroscopy (NIRS), a non-invasive and efficient optical imaging technique using the near-infrared region of the electromagnetic spectrum from 780 to 2,500 nm (4,000 to 12,820 cm^−1^), detects compositional changes in substances by utilizing the overtone and combination vibrations of CH, NH, and OH groups in molecules (Hirosawa et al., 2002; Huang et al., 2020; McIntosh et al., 2001), providing information about sample composition and chemical properties. Compared to commonly used high-throughput metabolomics analysis instruments such as mass spectrometry (MS), nuclear magnetic resonance (NMR), and chromatography analysis, NIRS analysis offers the advantages of convenience, ease of operation, a user-friendly interface, and the ability to analyze plasma without requiring sample preprocessing (Scalbert et al., 2009). Moreover, NIRS overcomes the common limitations of high cost and time consumption associated with these analytical instruments.

Our study employed NIRS-based analysis integrated with machine learning to differentiate MM from other plasma samples with high accuracy and specificity, as reflected in the area under the curve (AUC) values. Notably, we found that the support vector machine (*SVM*) and the partial least squares (*PLS*) algorithm demonstrated the best performance in both diagnosis and discriminant diagnosis. These results demonstrate strong clinical utility for efficient, convenient and precise diagnosis and discriminant diagnosis of MM. Our study demonstrates strong potential to improve the precision of MM diagnosis and discriminant diagnosis, particularly for rapid, large-scale screening in asbestos-exposed populations.

## Method

### Participants and plasma samples collection

**Figure 1:**
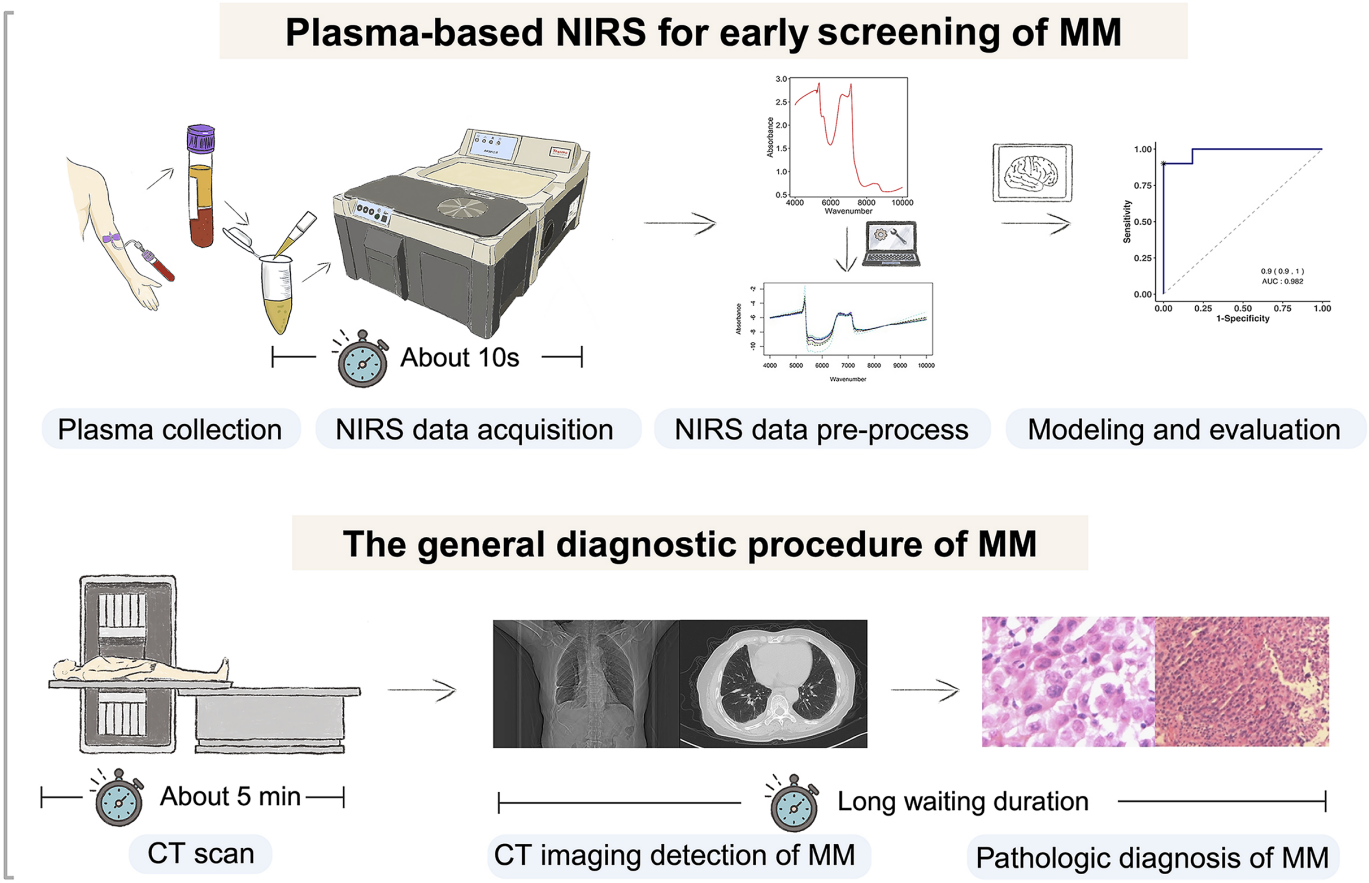
Flowchart of the study design. Our study involved selecting individuals with malignant mesothelioma (MM), lung cancer (LC), and gender and age-matched healthy controls. Plasma samples were collected and analyzed using near-infrared spectroscopy (NIRS) to obtain spectral data. Machine learning models were developed, trained, and evaluated using this data for predictive purposes. Created with BioRender.com.

Figure 1 depicts a graphical abstract summarizing the methodology employed in this study. The plasma samples analyzed in this study were obtained from Zhejiang Cancer Hospital (Hangzhou, China) between October 2016 and October 2023. These samples were collected from patients who had been diagnosed with the respective diseases between June 2016 and July 2023. A total of 99 plasma samples were collected, comprising 29 samples from pathologically diagnosed MM patients, 41 samples from pathologically diagnosed lung cancer (LC) patients, and 29 samples from healthy controls (HCs). Detailed clinical information of the participants is presented in Table 1. The collected samples were stored at −80 °C until analysis. All procedures involving human participants were conducted in accordance with the ethical standards set by the Ethics Committee of Zhejiang Cancer Hospital (IRB-2023-756), adhering to the principles outlined in the 1964 Helsinki Declaration and its subsequent amendments, or equivalent ethical standards. Prior to participating in the study, all participants provided informed consent for the utilization of their plasma samples. Furthermore, since the samples used in our study came from the biobank of Zhejiang Cancer Hospital, it was agreed that patient consent was not required.

**Table 1:** Detailed clinical information of the participants. A total of 99 plasma samples were collected, comprising 29 samples from pathologically diagnosed MM patients, 41 samples from pathologically diagnosed LC patients, and 29 samples from healthy controls (HCs). Detailed clinical information of the participants is presented in Table 1.

Feature	MM^a^ (*n* = 29)	LC^b^ (*n* = 41)	HC^c^ (*n* = 29)	*P*-value
Age				
Mean ± SD^d^	64.2 ± 8.8	61.6 ± 10.2	59.7 ± 6.7	NS^e^
Gender				
Male	17 (58.6%)	25 (70.0%)	14 (48.3%)	NS^f^
Female	12 (41.4%)	16 (30.0%)	15 (51.7%)	

**Notes:**

a Patient with malignant mesothelioma.

b Patient with lung cancer.

c Healthy control.

d Standard deviation.

e *P*-value was based on Wilcox test, *P*-value < 0.05 represents significant difference between two comparison groups.

f *P*-value was based on chi-square test, *P*-value < 0.05 represents significant difference between two comparison groups.

### NIR spectral data acquisition

The frozen samples were thawed on ice and gradually equilibrated to room temperature before analysis. After sample collection and prior to NIRS analysis, all plasma samples were visually inspected, and any samples exhibiting visible hemolysis (*e.g*., discoloration) were excluded from this study, which was identical to that used previously (Zhang et al., 2024). The NIR spectra of the plasma samples in our study were collected using an Antaris™ II FT-NIR analyzer (ThermoFisher Nicolet, Waltham, MA, USA) with air as a reference. A 6 mm pathlength colorimetric tube was used as the sample cup. Each spectrum was obtained from 30 successive scans from 4,000 to 10,000 cm^−1^ with a spectral resolution of 4 cm^−1^. The spectrum information was recorded by absorbance. For each sample, three replicate measurements (each comprising 30 scans) were acquired consecutively with the sample cup remaining inside the instrument; thus, no cleaning or emptying was performed between replicates. The average spectrum per sample was obtained using TQ Analyst 8.0 software.

### Data analysis

#### Spectral data random division

We utilized “*sample*” function in R to randomly divide the dataset consisted of absorbance values at different wavenumbers obtained from 99 plasma samples, including HC, LC, and MM samples, into two parts: a training set and a testing set. Among them, 66 plasma samples were included in the training set, including 18 HC samples, 29 LC samples and 19 MM samples. The testing set consisted of 33 plasma samples, including 11 HC samples, 12 LC samples, and 10 MM samples.

#### Spectral data pre-processing

Prior to pre-processing, we performed unsupervised principal component analysis (PCA) on the raw spectral data to explore its inherent structure and assess potential outliers. The PCA score plot is provided in Fig. S1. All samples were experimentally collected under standardized conditions, and no data points were excluded, as none were identified as outliers during PCA evaluation. Following this, wavenumbers with absorbance values exceeding 3 were excluded. To verify that key variables with high influence on principal component structure were not concentrated in noisy regions, we examined the loadings of the first principal component. The spectral regions with the highest absolute PC1 loadings were successfully excluded during the filtering process, as their absorbance values exceeded the threshold. The corresponding loading profile is shown in Fig. S2. The effect of different spectral pre-processing methods on machine learning (ML) model classification accuracy was evaluated. The pre-processing methods investigated were centering and scaling (CS), standard normal variate (SNV) (Adewale et al., 2014; Winkler-Moser et al., 2015), multiplicative scatter correction (MSC) (Zhang et al., 2020), first derivative (DERIV1), second derivative (DERIV2), standard normal variate and centering and scaling (SNV_CS), centering and scaling and standard normal variate (CS_SNV). Detailed information regarding each preprocessing method is provided in File S1.

We utilized these seven preprocessing methods alongside the raw data, constituting eight datasets for subsequent machine learning modeling and analysis. Additionally, the parameters of the chosen preprocessing method remain unchanged between the training and testing sets.

#### Spectral data modeling

For the data processed using the eight preprocessing methods we mentioned earlier, we further selected ten ML models to establish diagnostic and discriminant diagnostic models. Different combinations of two class categories were imported into the diagnostic and discriminant diagnostic models. In the case of importing the data into the ten ML algorithms for constructing the MM and LC discriminant diagnostic models, we excluded data from the HC class in both the training and testing sets for the preprocessing methods mentioned earlier, as well as the raw data, resulting in a total of eight preprocessing methods. Similarly, for constructing the MM diagnostic models, we excluded data from the LC class in both the training and testing sets, with the eight same preprocessing methods in total as well. To further demonstrate that models established in our study can be applied to different disease categories of plasma NIRS data, we also conducted an exploration using the same methodology on NIRS data from the class LC and class HC, evaluating combinations of 10 ML models and eight preprocessing methods we mentioned earlier across both training and testing sets.

We used ten ML algorithms, namely support vector machine (*SVM*), partial least squares (*PLS*), k-nearest neighbors (*KNN*), regularized random forest (*RRF*), parallel random forest (*ParRF*), ranger random forest (*Ranger*), random forest (*RF*), naive bayes (*NB*), classification and regression trees (*CART*), extreme gradient boosting (*XGBTREE*) respectively. All the modeling were conducted by using *caret* R package. The specific parameters of each ML model are provided in File S1.

Also, during the modeling process, due to the randomness of single random sampling for training set and testing set, models may perform well under specific random splits but poorly under others, limiting a comprehensive evaluation of model performance. To address this, we utilized the “*resamples()*” function in the *caret* R package to conduct cross-validation and repeated sampling, dividing the dataset into subsets for model training and testing. The resampling procedures include 3-fold cross-validation repeated 5 times, leave-one-out cross-validation, and bootstrapping. Models were evaluated in each subset, then metrics were stored, and finally, all evaluation results were aggregated for assessing each model. After setting up the modeling process with 3-fold cross-validation and five repetitions, the distribution of accuracy values and kappa values for each model under each pre-processing method were obtained for model assessment as well.

#### Models evaluation and optimized model selection

To identify the most suitable combination of preprocessing methods and ML algorithms for the research data at hand, we firstly evaluated the predictive performance of each model by comparing accuracy, sensitivity, specificity values in the training set respectively, as well as in the testing set. The accuracy is the overall classification accuracy of the model, which is calculated as [True Positive (TP) + True Negative (TN)]/[TP + TN + False Positive (FP) + False Negative (FN)]. The sensitivity, also known as recall, is calculated as TP/(TP + FN). The specificity is calculated as TN/(TN + FP). By considering these four metrics collectively, we will select an optimal combination of algorithm and preprocessing method to construct a model that can be utilized for the discriminant diagnosis of MM, as well as the diagnosis of MM.

To evaluate the performance of ML-preprocessing combinations for MM diagnosis and discriminant diagnosis, pairwise tests were conducted on resampled results of accuracy values of each combination from 3-fold cross-validation with 5 repetitions. Optimal combinations were selected based on mean differences, Cohen’s d, and *p*-values of pairwise tests.

#### ML models further evaluation by permutation test

After selecting the optimal ML-preprocessing combination, further permutation test is conducted on the chosen model to investigate the randomness of the model prediction results obtained after a single model run. The previously partitioned training set for distinguishing class LC *vs* class MM and class HC *vs* class MM is individually used to import the selected ML model for prediction based on the matching of each sample’s true class. Accuracy and Kappa values are obtained. Subsequently, the sequence of actual class matches for each sample in the training set is randomized using the “*sample()”* function in R, generating a new class label order for permutation test. This process is repeated 700 times, with each iteration yielding a set of acuracy and kappa values, thereby forming a permutation result distribution for comparison with values obtained under true class matching conditions. If the results under true class matching conditions deviate significantly from the permutation result distribution, the predictive performance of the model under true class matching conditions show low randomness, further validating the reliability of the model.

#### Feature selection

Due to the measurement principle of NIRS is based on the absorption of specific wavelengths of light by different functional groups and chemical bonds in a liquid matrix (Ren et al., 2020; Vieira et al., 2021), in order to further explore the features that make significant contributions to these classification models, and also further explore the functional groups that exhibit significant differences among various types of plasma, we analyzed the features in these classification models by conducting feature selection.

After selecting the most optimized ML model and pre-processing method combination for the data in our study, to differentiate which wavelengths in the NIRS data carry key and feature information about the different classes, there were two different feature selection approaches: Feature selection based on the variable importance in projection within the *PLS* model (PLS-VIP), and the support vector machine recursive feature elimination (SVM-RFE) algorithm based feature selection for the *SVM* model. The variable importance in projection (VIP) score is utilized to assess the significance of each variable employed in the *PLS* model (Wang, He & Wang, 2015). The SVM-RFE is a wrapper feature selection method which generates the ranking of features using backward feature elimination (Huang et al., 2014). The features are eliminated according to a criterion related to their support to the discrimination function, and the *SVM* is re-trained at each step.

## Result

### NIR spectral characteristics of plasma samples

A total of 99 plasma samples, consisting of HC, LC, and MM groups, were subjected to NIRS analysis at intervals of every four wavenumbers within the wavenumber range of 4,000 to 10,000 cm^−1^. Since each collected wavenumber represents a candidate variable for building the ML model, wavenumber features exceeding a threshold of 3 in any sample were excluded, we ultimately used 1,291 variables for constructing both the diagnostic model and the discriminant diagnostic model.

### Selecting the optimal combination of spectral pre-processing method and ML algorithms

**Figure 2:**
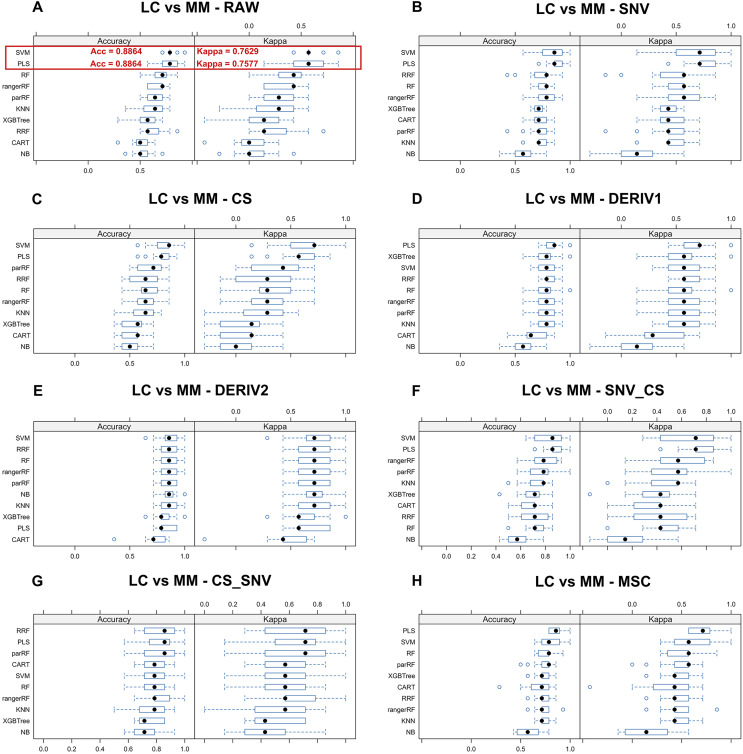
Predictive performance of MM discriminant diagnostic model in training set. Incorporating eight preprocessing methods, including the (A) raw data, (B) centering and scaling (CS), (C) second derivative processing, (D) centering and scaling and standard normal variate (CS_SNV), (E) standard normal variate (SNV), (F) first derivative processing, (G) standard normal variate and centering and scaling (SNV_CS), (H) multiplicative scatter correction (MSC), with 10 ML algorithms including support vector machine (*SVM*), partial least squares (*PLS*), k-nearest neighbors (*KNN*), regularized random forest (*RRF*), parallel random forest (*ParRF*), ranger random forest (*Ranger*), random forest (*RF*), naive bayes (*NB*), classification and regression trees (*CART*), extreme gradient boosting (*XGBTREE*), each MM discriminant diagnostic model’s accuracy and kappa value distributions are visualized in A–H Whisker plots after 3-fold cross-validation repeated 5 times for resampling process.

**Figure 3:**
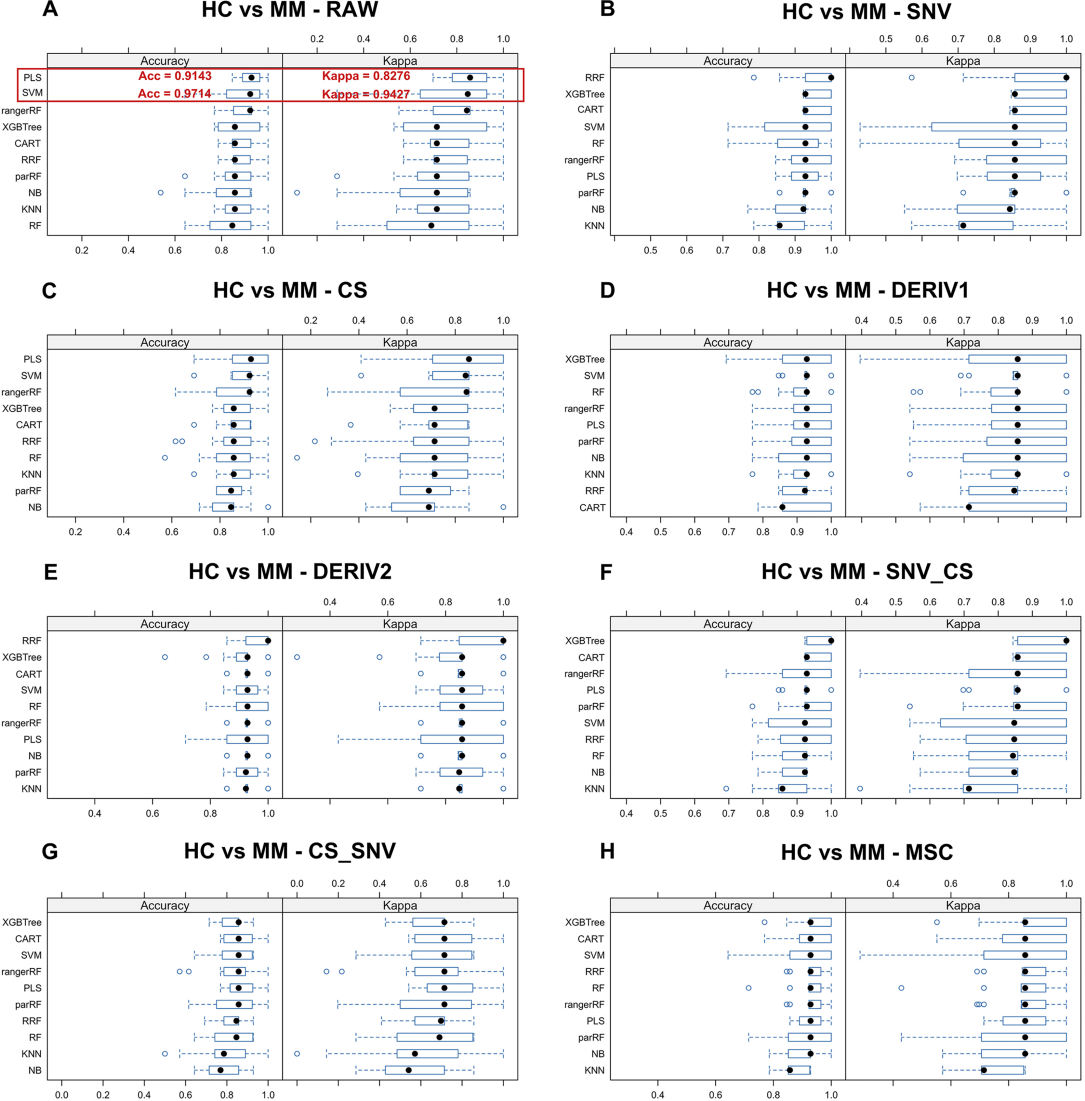
Predictive performance of MM diagnostic model in testing set. Incorporating eight preprocessing methods, including the (A) raw data, (B) centering and scaling (CS), (C) second derivative processing, (D) centering and scaling and standard normal variate (CS_SNV), (E) standard normal variate (SNV), (F) first derivative processing, (G) standard normal variate and centering and scaling (SNV_CS), (H) multiplicative scatter correction (MSC), with 10 ML algorithms, each MM diagnostic model’s accuracy and kappa value distributions are visualized in A–H whisker plots after 3-fold cross-validation repeated 5 times for resampling process.

We aim to identify the optimal pre-processing method and the ML algorithm combination with the best predictive performance from various combinations which consists of eight preprocessing methods, including raw data, and ten ML algorithms. Pre-processing methods can be observed in Fig. S3 to visualize the changes in the distribution patterns of spectral data on a two-dimensional plane. The red line in each plot represents the mean NIR spectrum of all MM samples at each wavenumber after each pre-processing method. Whisker plots showing the distribution of accuracy and kappa results generated by each model training and predicting after the resampling process using training set data for distinguishing class LC and class MM or for distinguishing class MM and class HC, repeated 5 times with 3-fold cross-validation (Figs. 2 and 3). Using the same presentation format, Fig. S2 displays the corresponding results for the models distinguishing class LC and class HC. Accuracy is a measure of a classification model’s precision in predictions, while Kappa evaluates the consistency between a model’s predictions and actual observations. Therefore, it is evident that the combination of raw data with *PLS* or *SVM* ML algorithms yielded significantly better results in the training set compared to other ML-preprocessing combinations. In models for MM discriminant diagnosis, the *PLS* model achieved an accuracy of 0.8863 and a kappa value of 0.7577, while the *SVM* model achieved an accuracy of 0.8863 and a kappa value of 0.7692. In models distinguishing class HC and class MM, the *PLS* model had an accuracy of 0.9142 and a kappa value of 0.8276, whereas the *SVM* model had an accuracy of 0.9705 and a kappa value of 0.9427. The accuracy values of model predictions on the training set for each model obtained by combining eight pre-processing methods, including raw data, with ten ML algorithms can be found in Table 2, which also indicates that these classification models constructed by combining raw data with *PLS* or *SVM* ML algorithms exhibit significantly superior predictive results in the training set compared to combinations of other preprocessing methods and ML algorithms. Predictive performance of all models in training set and testing set can be found in Tables S2 and S4 respectively. In addition, the predictive performance of models for distinguishing class LC and class HC on the training set and testing set in details can be seen in the Tables S5 and S6. The combination of *SVM* and *PLS* with raw data demonstrates strong predictive capabilities in distinguishing between HC and LC categories across both datasets.

**Table 2:** Preprocessing results for ten types of ML algorithms on the training set. The accuracy values of model predictions on the training set for each model obtained by combining eight preprocessing methods, including raw data, with ten ML algorithms can be found in Table 2, which also indicates that these classification models constructed by combining raw data with *PLS* or *SVM* ML algorithms exhibit significantly superior predictive results in the train set compared to combinations of other preprocessing methods and ML algorithms.

Group	Pre-processing method	Training set accuracy									
		SVM^a^	PLS^b^	KNN^c^	RF^d^	NB^e^	ParRF^f^	Ranger^g^	CART^h^	XGBTree^i^	RRF^j^
LC *vs* MM	No pre-processing	0.8864	0.8864	0.6818	0.8864	0.5682	0.8636	0.8864	0.6042	0.7273	0.7727
	SNV^k^	0.9318	0.9091	0.7727	0.9318	0.7500	0.9318	0.9773	0.8409	0.9545	0.9318
	MSC^l^	0.9773	0.9091	0.8182	0.9091	0.8182	0.8864	0.8864	0.8409	0.8636	0.9091
	CS^m^	0.8864	0.8864	0.6364	0.8182	0.6364	0.8409	0.8636	0.6364	0.6364	0.8636
	SNV+CS^n^	0.6136	0.8834	0.4318	0.6364	0.3864	0.3864	0.3864	0.3864	0.3864	0.3636
	CS+SNV^o^	0.9318	0.8636	0.8636	0.9091	0.9318	0.8864	0.9091	0.8864	0.8864	0.8182
	1^st^ Der^p^	0.9318	0.8864	0.7955	0.9545	0.8182	0.9545	0.9545	0.7955	0.9545	0.9545
	2^nd^ Der^q^	0.9545	0.8864	0.8864	0.9545	0.9091	0.9545	0.9545	0.8864	0.9545	0.9545
HC *vs* MM	No pre-processing	0.9714	0.9143	0.7714	0.9143	0.8000	0.9143	0.9143	0.8571	0.8857	0.9143
	SNV	1.0000	0.9143	0.8000	0.9429	0.8000	0.9429	0.9714	0.9714	0.9714	0.9714
	MSC	1.0000	0.9143	0.8000	0.9429	0.8000	0.9429	0.9429	0.9429	0.9429	0.9429
	CS	0.9714	0.9429	0.7714	0.8571	0.7714	0.8286	0.8571	0.8286	0.8857	0.8857
	SNV+CS	0.4857	0.8857	0.5714	0.6285	0.4857	0.8571	0.6857	0.8571	0.8571	0.8571
	CS+SNV	0.9429	0.8857	0.8571	0.9714	0.8000	0.9714	0.9714	0.9429	0.9714	0.9429
	1^st^ Der	0.9714	0.9143	0.8286	0.9714	0.8571	0.9714	0.9714	0.9714	0.9714	0.9714
	2^nd^ Der	0.9714	0.9714	0.9143	1.0000	0.9143	0.9714	1.0000	0.9714	0.9714	1.0000

**Notes:**

a Support Vector Machines.

b Partial Least Squares.

c K-Nearest Neighbors.

d Random Forest.

e Naive Bayes.

f Parallel random forest.

g Ranger random forest.

h Classification and regression trees.

i Extreme gradient boosting.

j Regularized random forest.

k Standard normal variate.

l Multiplicative scatter correction.

m Centering and scaling.

n The preprocessing method combining standard normal variate followed by centering and scaling.

o The preprocessing method combining centering and scaling followed by standard normal variate.

p First derivative.

q Second derivative.

Pairwise tests revealed the consistent superiority of *SVM* and *PLS* among all ML-preprocessing combinations for MM diagnosis and discriminant diagnosis. Specifically, *SVM* with raw data excelled in discriminant diagnosis (*P* = 1.99 × 10^−7^, mean difference = 0.29, Cohen’s d = 3.55), while *PLS* with raw data performed best in diagnosis (*P* = 0.006, mean difference = 0.11, Cohen’s d = 1.19) (Table S7).

Based on these results and the requirements for fast screening, raw data were selected for constructing the final diagnostic and discriminative models due to their minimal statistical processing requirements and operational simplicity.

### Permutation test of the models

**Figure 4:**
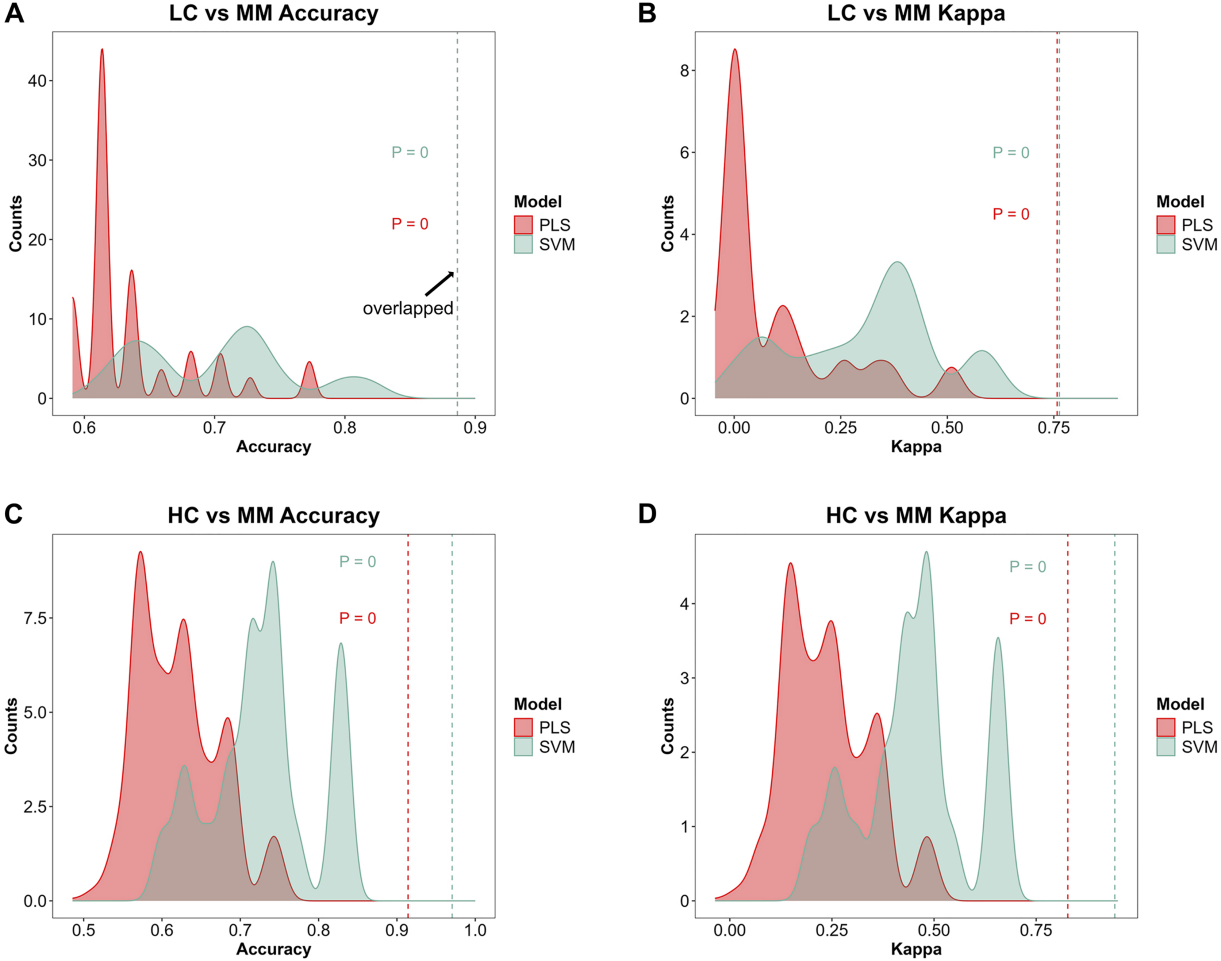
Model further evaluation by permutation test. After 700 times of permutation tests, the distribution of permutation test results and their comparison with the model predictions based on the true class labels is shown. The red area and red dashed line represent the permutation test distribution results and the prediction performance with the true class labels of the *PLS* model respectively. The green area and green dashed line represent those in *SVM* model respectively. In the MM discriminant diagnostic models, (A) the accuracy of models with true class label is 0.8864 in both *PLS* and *SVM*. (B) The kappa value of models with true class label is 0.7577 in *PLS* and 0.7629 in *SVM*. In the MM diagnostic models, (C) the accuracy of models with true class label is 0.9143 in *PLS* and 0.9714 in *SVM*. (D) The kappa value of models with true class label is 0.8276 in *PLS* and 0.9427 in *SVM*.

Figures 4A–4D demonstrate the distribution of accuracy and kappa values after 700 times of permutation test for *PLS* and *SVM* models in distinguishing class HC and class MM or these models in distinguishing class LC and class MM, compared with the accuracy and kappa values of the models under the true class labels corresponding to each sample. The dashed lines in the Figs. 4A–4D indicate the accuracy or kappa values of the models under the true class labels corresponding to each sample. All dashed lines deviate from the distribution range of accuracy or kappa values obtained after permutation test, demonstrating the credibility of the classification models used in our study.

### Feature selection of the models

The absorption characteristics of near-infared by different molecular chemical bonds and functional groups in samples are utilized by NIR to infer the composition and structural details of the samples. By ranking the importance of features in the optimal diagnostic and discriminant diagnostic models, distinct wavenumber ranges with significant spectral differences between contrasting sample groups during NIRS measurements are identified, allowing for the inference of potential differences in functional groups and chemical bonds.

**Figure 5:**
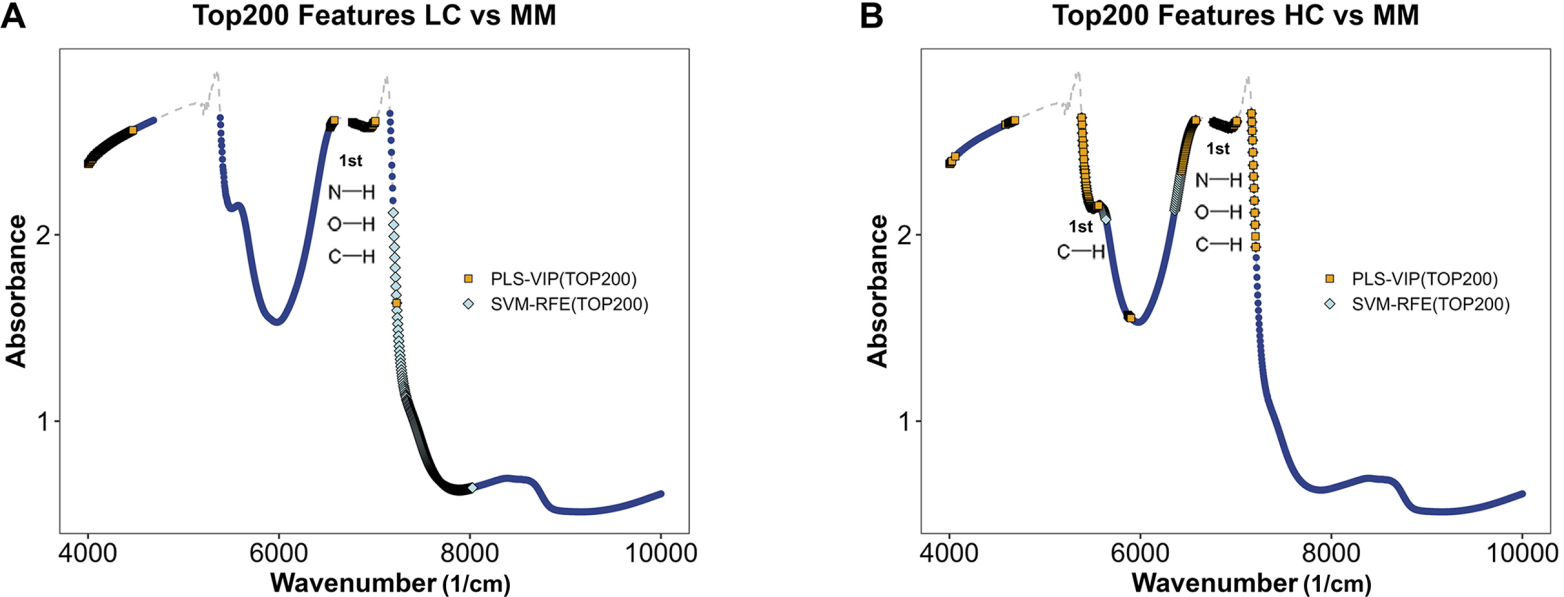
Feature selection in MM discriminant diagnostic model and MM diagnostic model. The MM spectrum after filtering features with absorbance exceeding 3 in any sample, highlighting the top 200 features selected by PLS-VIP and SVM-RFE. Gray dashed lines indicate excluded regions. (A) In the MM discriminant diagnostic models, the features obtained from both methods are concentrated in the spectral regions of 7,197–7,231 cm^−1^. (B) In the MM diagnostic models, the features obtained from both methods are primarily concentrated in the spectral regions of 5,380–5,560, 6,430–6,529 and 6,765–7,208 cm^−1^.

Figures 5A and 5B illustrate the distribution of models for distinguishing class LC and class MM or class HC and class MM after integrating preprocessing methods with *PLS* and *SVM*. By using feature selection methods *via* PLS-VIP and SVM-RFE, the figure highlights segments in different colors on the full spectral curve for each class, indicating the distribution of the top 200 ranked features on MM spectra after feature ranking through PLS-VIP and SVM-RFE methods. We observe that in the models aimed at distinguishing LC and MM, the top 200 features selected by PLS-VIP are predominantly distributed in the ranges of 4,000–4,500 and 6,500–7,200 cm^−1^, while those selected by SVM-RFE are mainly in the range of 7,200–8,000 cm^−1^. In the distinguishing class HC and class MM, the top 200 features selected by PLS-VIP are mainly distributed in the ranges of 4,000–4,100, 4,600–4,700, 5,400–5,600, 6,400–6,500 and 6,700–7,200 cm^−1^, whereas those selected by SVM-RFE are predominantly in the ranges of 5,400–5,600, 6,700–7,200 and 6,300–6,600 cm^−1^. Referring to Table 3, the range of 5,400–5,600 cm^−1^ approximately corresponds to the first overtones of CH, and the range of 6,400–7,200 cm^−1^ roughly corresponds to the first overtones of OH, CH, and NH. These results suggest potential differences in functional groups between different types of plasma samples.

**Table 3:** The NIR spectral peaks and their tentative assignments of functional groups.

Wavenumber (cm^−1^)	Assignments (Weyer, 1985; Ben-Dor, 1997; Bokobza, 1998)
4,200–5,500	The CH, OH and NH stretch/CH deformations in the phenyl
5,130–5,230	The second overtone of CO
5,710–5,780	The first overtone of SH
5,400–6,100	The first overtones of the CH stretch vibrations
6,200–7,600	The first overtone of OH, NH, and CH
7,900–9,000	The second overtone of CH. NH, and CH combinations
8,850–10,000	The second overtone of OH combination

### Prediction performance of optimized models on the testing set

**Figure 6:**
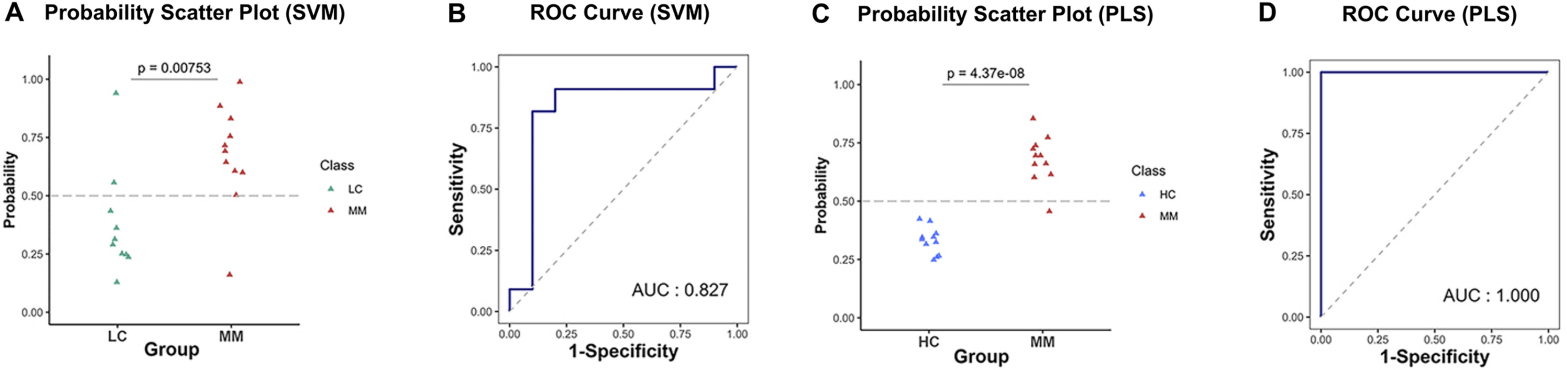
Predictive performance in MM discriminant diagnostic model and MM diagnostic model on the testing set. (A) In MM discriminant diagnostic model, the scatter plot of probability values in each class of *SVM* model. (B) In MM discriminant diagnostic model, the ROC curve of *SVM* model, with the AUC value of 0.827. (C) In MM diagnostic model, the scatter plot of probability values of *PLS* model. (D) In MM diagnostic model, the ROC curve of *PLS* model, with the AUC value reached 1.

Table 4 presents the predictive performance results of models combining eight preprocessing methods and 10 ML algorithms in the diagnostic and discriminant diagnostic for MM using testing set. After selecting the optimal preprocessing methods and ML algorithms, we have obtained models combining the raw data with *PLS* and *SVM*, which are most suitable for both diagnosis and discriminant diagnosis of MM. Then we applied the testing set to these models to evaluate their predictive performance. Figures 6A–6D illustrate the predictive performance of the models when the testing dataset is applied through receiver operating characteristic (ROC) curves and probability distribution scatter plots. The probability value distributions of each model in each group exhibit significant differences, and the AUC values of each model are close to 1, demonstrating strong predictive performance of these models. The *SVM*–raw data combination achieved optimal performance for MM discriminant diagnosis (AUC = 0.827), while the *PLS*–raw data combination performed best for MM diagnosis (AUC = 1.0). Predictive performance of these models on the testing set in details can be found in the Table S1 and the Table S3.

**Table 4:** Preprocessing results for ten types of ML algorithms on the testing set. The accuracy values of model predictions on the testing set for each model obtained by combining eight preprocessing methods, including raw data, with ten ML algorithms can be found in Table 4, which also indicates that these classification models constructed by combining raw data with *PLS* or *SVM* ML algorithms exhibit significantly superior predictive results in the testing set compared to combinations of other preprocessing methods and ML algorithms.

Group	Pre-processing method	Testing set accuracy									
		SVM^a^	PLS^b^	KNN^c^	RF^d^	NB^e^	ParRF^f^	Ranger^g^	CART^h^	XGBTree^i^	RRF^j^
LC *vs* MM	No pre-processing	0.9524	0.8571	0.8095	0.9524	0.6190	0.9524	0.9524	0.5714	0.8571	0.7619
	SNV^k^	1	0.8571	0.7619	0.9524	0.6190	0.9524	0.9524	0.8571	0.9524	0.9524
	MSC^l^	0.5238	0.8571	0.4286	0.5238	0.5238	0.5238	0.5238	0.4762	0.5238	0.5238
	CS^m^	0.9524	0.8095	0.4762	0.5238	0.6667	0.5238	0.5714	0.5238	0.6190	0.5714
	SNV+CS^n^	0.5238	0.4762	0.5238	0.5238	0.5238	0.5238	0.5238	0.4762	0.5238	0.5238
	CS+SNV^o^	1	0.7619	0.8571	0.9048	0.6667	0.8571	0.8571	0.7619	0.8571	0.7619
	1^st^ Der^p^	0.9524	0.8571	0.8095	0.9524	0.8095	0.9524	0.9524	0.9524	0.9524	0.9524
	2^nd^ Der^q^	0.9524	0.9571	0.8095	0.9524	0.8095	0.9524	0.9524	0.8571	0.9524	0.9524
HC *vs* MM	No pre-processing	0.9545	0.9545	0.7727	0.8636	0.8182	0.8636	0.8636	0.8182	0.8636	0.8182
	SNV	1.0000	0.9545	0.9091	1.0000	0.9091	1.0000	1.0000	1.0000	1.0000	1.0000
	MSC	0.9545	0.9545	0.8182	0.9545	0.5000	0.9545	0.9545	0.9545	0.9545	0.9545
	CS	1.0000	0.9545	0.7727	0.8636	0.7727	0.8636	0.8636	0.8636	0.8636	0.8636
	SNV+CS	0.5000	0.5000	0.5000	0.5000	0.5000	0.5000	0.5000	0.5000	0.5000	0.5000
	CS+SNV	1.0000	0.9091	0.9091	1.0000	0.8636	1.0000	1.0000	0.9545	0.9545	0.9545
	1^st^ Der	1.0000	0.9545	0.9524	0.9545	0.9545	1.0000	1.0000	1.0000	1.0000	0.9545
	2^nd^ Der	0.9545	1.0000	0.9545	1.0000	1.0000	1.0000	1.0000	1.0000	1.0000	1.0000

**Notes:**

a Support Vector Machines.

b Partial Least Squares.

c K-Nearest Neighbors.

d Random Forest.

e Naive Bayes.

f Parallel random forest.

g Ranger random forest.

h Classification and regression trees.

i Extreme gradient boosting.

j Regularized random forest.

k Standard normal variate.

l Multiplicative scatter correction.

m Centering and scaling.

n The preprocessing method combining standard normal variate followed by centering and scaling.

o The preprocessing method combining centering and scaling followed by standard normal variate.

p First derivative.

q Second derivative.

## Discussion

In China, the diagnosis and differentiation of MM remain challenging, especially in primary care settings. There is a critical need for rapid and convenient diagnostic methods prior to CT imaging. Current blood-based tumor markers show limited accuracy; for instance, the loss of BAP-1 used to distinguish MM from lung cancer which exhibits highly variable sensitivity (55–85%) despite high specificity (98–100%) (Fels Elliott & Jones, 2020).

To address this, we developed a novel approach combining *PLS* and *SVM* machine learning algorithms with non-invasive, rapid NIRS analysis of raw spectral data.

NIRS offers multiple advantages over proteomic or genomic techniques (Eberhardt et al., 2015; Ferrari, Mottola & Quaresima, 2004; Scalbert et al., 2009), including non-invasiveness, low cost, ease of operation, and rapid analysis. Specifically, it requires no sample pretreatment, enabling direct *in situ* measurement. Its operational principle is straightforward, minimizing training requirements and facilitating use in primary care institutions. Additionally, NIRS provides high-throughput capability with short per-sample acquisition time, significantly reducing overall analytical time and cost. These attributes make it particularly suitable for large-scale screening applications, such as profiling plasma samples.

The diagnostic and discriminant diagnostic models developed in our study integrate support vector machine and partial least squares algorithms with NIRS data from categorized plasma samples. These models demonstrate high accuracy, specificity, and sensitivity, along with low rates of misdiagnosis and missed diagnosis. In terms of computational efficiency, *SVM* and *PLS* algorithms required shorter processing times than other models. Among the random forest algorithms evaluated, *rangerRF* showed the highest computational efficiency, while *RRF* was the most time-consuming. For spectral preprocessing, SNV and DERIV2 improved predictive performance across most models, particularly those other than *SVM* and *PLS*. The sequence of centering followed by scaling and SNV achieved superior results in both diagnostic and discrimination models compared to the sequence of SNV followed by centering then scaling. Furthermore, feature selection consistently identified the spectral region between 6,530 and 7,200 cm^−1^, which corresponds to the first overtones of OH, NH, and CH stretching vibrations, among the most relevant features in all four models. This consistent selection suggests meaningful biochemical differences related to these functional groups among the three plasma sample classes.

In conclusion, our study developed a non-invasive, low-cost, and convenient plasma analysis method using NIRS and ML for rapid and discriminative diagnosis of MM. The approach achieves high accuracy by directly processing raw spectral data from plasma samples, offering a fast and efficient screening solution, especially when employed for large-scale rapid screening of populations with MM in areas with high-level asbestos exposure at the primary care institution.

This method serves as an effective tool for initial identification of suspected malignant thoracic tumors among patients with respiratory symptoms, enabling timely referral to specialized centers for further diagnosis. It also aids in distinguishing MM from lung cancer (LC), providing critical support for discriminant diagnosis of MM. By improving classification accuracy, the model facilitates better clinical decision-making and paves the way for more personalized treatment strategies.

Looking forward, this approach established in our study can be integrated with assessments by radiologists and pathologists to enhance diagnostic reliability. Patients flagged by this rapid screen can undergo subsequent pathological confirmation, reducing misdiagnosis risks and treatment delays, ultimately improving outcomes for those with rare thoracic malignancies.

Our study also has several limitations. First, the sample size was relatively small and sourced from a single center, constrained by the rarity of MM, which may limit the statistical power and generalizability of our NIRS model. Due to the rarity of MM and the lack of accessible, comparable plasma-based NIR datasets, independent external validation could not be performed in this study. We acknowledge this limitation and plan to address it in future work through multi-center collaborations and larger prospective studies. Second, the absence of detailed CT data from the same cohort hindered direct comparison between NIRS and radiographic findings; future studies should incorporate such correlative analyses to enhance diagnostic interpretation. Furthermore, given that NIRS primarily detects functional groups rather than specific molecules, quantitative metabolic profiling remains challenging. Future study could integrate mass spectrometry-based metabolomics to elucidate compositional differences among disease groups more precisely.

## Conclusion

In conclusion, our study established a novel, non-invasive platform integrating NIRS with ML, which set a foundation and highlighted potential opportunities for future large-scale plasma screening of MM and discriminant of MM, particularly among populations with high asbestos exposure.

## Supplemental Information

10.7717/peerj.20503/supp-1 Supplemental Information 1 Raw data. All samples and the absorbance values of each sample at each wavenumber.

10.7717/peerj.20503/supp-2 Supplemental Information 2 Code used for data analysis throughout this study.

10.7717/peerj.20503/supp-3 Supplemental Information 3 Translations of Chinese text in code.

10.7717/peerj.20503/supp-4 Supplemental Information 4 PCA score plot of the raw NIR spectral data, used to evaluate data structure and detect potential outliers. No samples were excluded based on this assessment.

10.7717/peerj.20503/supp-5 Supplemental Information 5 Loadings of the first principal component (PC1) across all wavenumbers in the raw spectral dataset, used to assess the influence of noisy regions.

10.7717/peerj.20503/supp-6 Supplemental Information 6 Predictive performance of LC diagnostic model in training set. Incorporating 8 preprocessing methods, including the (A) raw data, (B) centering and scaling(CS), (C) second derivative processing, (D) centering and scaling and standard normal variate (CS_SNV), (E) standard normal variate (SNV), (F) first derivative processing, (G) standard normal variate and centering and scaling (SNV_CS), (H) multiplicative scatter correction (MSC), with 10 ML algorithms, each LC diagnostic model’s accuracy and kappa value distributions are visualized in A–H Whisker plots after 3-fold cross-validation repeated 5 times for resampling process.

10.7717/peerj.20503/supp-7 Supplemental Information 7 Comparative visualization of raw and preprocessed near-infrared spectroscopy (NIRS) waveforms from mesothelioma (MM) plasma samples. Preprocessing steps include: (a) raw data, (b) standard normal variate (SNV) normalization, (c) Centering and scaling (CS), (d) standard normal variate (SNV) normalization and then c entering and scaling (CS), (e)multiplicative scatter correction (MSC), (f) c entering and scaling (CS) and then standard normal variate (SNV) normalization, (g) 1st derivative and (h) 2nd derivative.

10.7717/peerj.20503/supp-8 Supplemental Information 8 Predictive performance of HC *vs* MM on the testing set.

10.7717/peerj.20503/supp-9 Supplemental Information 9 Predictive performance of HC *vs* MM on the training set.

10.7717/peerj.20503/supp-10 Supplemental Information 10 Predictive performance of LC *vs* MM on the testing set.

10.7717/peerj.20503/supp-11 Supplemental Information 11 Predictive performance of LC *vs* MM on the testing set.

10.7717/peerj.20503/supp-12 Supplemental Information 12 Predictive performance of HC *vs* LC on the testing set.

10.7717/peerj.20503/supp-13 Supplemental Information 13 Predictive performance of HC *vs* LC on the training set.

10.7717/peerj.20503/supp-14 Supplemental Information 14 Pairwise test results based on each preprocessing-ML combination in training set.

## Additional Information and Declarations

### Data Availability

The following information was supplied regarding data availability:

The raw data are available in the Supplemental Files.
