# Supplementary material for: Near-infrared spectroscopy combined with machine learning for plasma-based discriminant diagnosis of malignant mesothelioma: a retrospective study
Source: PeerJ. 2025 Dec 19;13:e20503. doi: 10.7717/peerj.20503 (PMC12721102; doi:10.7717/peerj.20503)
Supplement: Supplemental Information 3 [file peerj-13-20503-s003.docx]

# Translations of Non-English Text in CODE.R

Line 16:

Original: 提取波数-Mac里正常显示，所以2,8改成1,7就行

Translation: Extract wavenumbers – displays correctly on Mac, so change 2,8 to 1,7.

Line 19:

Original: 去3

Translation: Remove spectra with absorbance > 3.

Line 24:

Original: 通过sample函数，获取train序列

Translation: Generate training sequence using the sample() function.

Line 29:

Original: 分别获取HCMM和LCMM以及HCLC的train和test的矩阵

Translation: Extract training and testing matrices for HCMM, LCMM, and HCLC groups.

Line 37:

Original: 定义预处理组合函数

Translation: Define preprocessing combination function.

Line 44:

Original: 定义SNV函数 Standard Normal Variate

Translation: Define SNV function (Standard Normal Variate).

Line 51:

Original: 定义CS函数 Center and Scale

Translation: Define CS function (Center and Scale).

Line 59:

Original: 定义The first derivative

Translation: Define the first derivative function.

Line 68:

Original: 定义The second derivative

Translation: Define the second derivative function.

Line 77:

Original: SNV + CS 组合

Translation: Combination of SNV and CS.

Line 84:

Original: CS + SNV 组合

Translation: Combination of CS and SNV.

Line 92:

Original: data.frame没有sample和class

Translation: The data.frame lacks 'sample' and 'class' columns.

Line 98:

Original: NIR处理-便于MSC,D2,D1预处理

Translation: NIR processing – prepare for MSC, D1, D2 preprocessing.

Line 102:

Original: 定义MSC Multiple Scattering Correction

Translation: Define MSC function (Multiplicative Scatter Correction).

Line 109:

Original: 转化D1 D2数据格式和其他数据一样,sample,class,var1,var2,var3.....varn.

Translation: Format D1/D2 data to match structure: sample, class, var1, var2, ..., varn.

Line 127:

Original: 对LCMM和HCMM以及HCLC进行预处理

Translation: Apply preprocessing to LCMM, HCMM, and HCLC.

Line 130:

Original: 取出train,test

Translation: Extract training and testing sets.

Line 134:

Original: 4000-10000波数不同预处理画图

Translation: Plot spectra under different preprocessing methods (4000–10000 cm⁻¹).

Line 135:

Original: 用train里边的跑

Translation: Run using training data.

Line 167:

Original: matplot方式

Translation: Using matplot method.

Line 184:

Original: 设置二分类模型

Translation: Set up binary classification model.

Line 185:

Original: # 5-fold CV, 10 repeats #需要根据情况修改

Translation: 5-fold cross-validation with 10 repeats (modifiable).

Line 198:

Original: predict(PLS,testdata,type = "prob") type= raw or prob ##用这个prob才是概率值

Translation: Use predict(PLS, testdata, type='prob') to get probability output.

Line 199:

Original: 得到allinfo均用confusionMatrix(predict(PLS,testdata[,-c(1:2)]),as.factor(testdata$Class))

Translation: Generate allinfo using confusionMatrix().

Line 214:

Original: # predict(SVM1,testdata[,-c(1:2)], probability = TRUE) %>% attributes() %>% .[["probabilities"]] prob被赋予属性形式存在，所以需要读取属性，再调用

Translation: SVM1: Probabilities stored as attributes – need to extract manually.

Line 220:

Original: predict(SVM2,testdata,type = "prob") caret内无法获取prob

Translation: SVM2: caret package does not support probability output.

Line 221:

Original: 得到allinfo均用confusionMatrix(predict(SVM2,testdata[,-c(1:2)]),as.factor(testdata$Class))

Translation: Generate allinfo using confusionMatrix().

Line 232:

Original: predict(NB2,testdata,type = "prob") type= raw or prob ##用这个prob才是概率值

Translation: NB2: Use predict with type='prob' for probabilities.

Line 233:

Original: 得到allinfo均用confusionMatrix(predict(NB,testdata[,-c(1:2)]),as.factor(testdata$Class))

Translation: Generate allinfo using confusionMatrix().

Line 245:

Original: predict(KNN,testdata,type = "prob") type= raw or prob ##用这个prob才是概率值

Translation: KNN: Use type='prob' to obtain probability values.

Line 246:

Original: 得到allinfo均用confusionMatrix(predict(KNN,testdata[,-c(1:2)]),as.factor(testdata$Class))

Translation: Generate allinfo using confusionMatrix().

Line 249:

Original: RF默认

Translation: Default Random Forest (RF) settings.

Line 255:

Original: predict(RF_default,testdata,type = "prob") type= raw or prob ##用这个prob才是概率值

Translation: Use predict(RF_default, testdata, type='prob') to get probability output.

Line 256:

Original: 得到allinfo均用confusionMatrix(predict(RF_default,testdata[,-c(1:2)]),as.factor(testdata$Class))

Translation: Generate allinfo using confusionMatrix().

Line 259:

Original: 并行RF算法

Translation: Parallel version of RF algorithm.

Line 265:

Original: predict(RF_par,testdata,type = "prob") type= raw or prob ##用这个prob才是概率值

Translation: Use predict(RF_par, testdata, type='prob') to get probability output.

Line 266:

Original: 得到allinfo均用confusionMatrix(predict(RF_par,testdata[,-c(1:2)]),as.factor(testdata$Class))

Translation: Generate allinfo using confusionMatrix().

Line 275:

Original: predict(RF_ranger,testdata)目前没找到概率值

Translation: RF_ranger: probability output not currently supported.

Line 276:

Original: 得到allinfo均用confusionMatrix(predict(RF_ranger,testdata[,-c(1:2)]),as.factor(testdata$Class))

Translation: Generate allinfo using confusionMatrix().

Line 286:

Original: predict(RF_regular,testdata,type = "prob") type= raw or prob ##用这个prob才是概率值

Translation: Use predict(RF_regular, testdata, type='prob') to get probability output.

Line 287:

Original: 得到allinfo均用confusionMatrix(predict(RF_regular,testdata[,-c(1:2)]),as.factor(testdata$Class))

Translation: Generate allinfo using confusionMatrix().

Line 298:

Original: predict(TREE,testdata,type = "prob") type= raw or prob ##用这个prob才是概率值

Translation: Use predict(TREE, testdata, type='prob') to get probability output.

Line 299:

Original: 得到allinfo均用confusionMatrix(predict(TREE,testdata[,-c(1:2)]),as.factor(testdata$Class))

Translation: Generate allinfo using confusionMatrix().

Line 328:

Original: predict(XGBTREE,testdata,type = "prob") type= raw or prob ##用这个prob才是概率值

Translation: Use predict(XGBTREE, testdata, type='prob') to get probability output.

Line 329:

Original: 得到allinfo均用confusionMatrix(predict(XGBTREE,testdata[,-c(1:2)]),as.factor(testdata$Class))

Translation: Generate allinfo using confusionMatrix().

Line 336:

Original: 到时候在说

Translation: To be discussed later.

Line 360:

Original: 基于train建模

Translation: Model training based on training set.

Line 361:

Original: #这一步选好需要进行的对比的数据

Translation: Select data for model comparison.

Line 362:

Original: #这一步选好需要用到的model

Translation: Select models to include.

Line 366:

Original: 去3

Translation: Remove spectra with absorbance > 3.

Line 367:

Original: #这一步选好需要进行的对比的数据

Translation: Select data for model comparison.

Line 368:

Original: #这一步选好需要用到的model

Translation: Select models to include.

Line 374:

Original: 去3+去异常值#

Translation: Remove spectra with absorbance > 3 and outliers.

Line 375:

Original: #这一步选好需要进行的对比的数据

Translation: Select data for model comparison.

Line 376:

Original: #这一步选好需要用到的model

Translation: Select models to include.

Line 383:

Original: 重新整理模型顺序

Translation: Reorganize model order.

Line 394:

Original: lapply方法

Translation: Using lapply method.

Line 404:

Original: 循环方法

Translation: Using loop method.

Line 430:

Original: 获取pvalue,byclass和overall信息

Translation: Obtain p-value, by-class and overall metrics.

Line 433:

Original: 使用数据框而不是向量

Translation: Use data frame instead of vector.

Line 446:

Original: 检查模型类型并相应处理

Translation: Check model type and handle accordingly.

Line 448:

Original: 对于PLS模型，需要确保测试数据与训练数据有相同的列

Translation: For PLS models, ensure test set has the same columns as training set.

Line 449:

Original: 获取训练时的列名

Translation: Retrieve column names from training set.

Line 452:

Original: 确保测试数据有相同的列（按相同的顺序）

Translation: Ensure test set has same column order.

Line 456:

Original: 进行预测

Translation: Perform prediction.

Line 458:

Original: 将预测结果转换为类别（根据你的具体需求调整）

Translation: Convert predictions to class labels (adjust as needed).

Line 459:

Original: 示例，请根据实际情况调整

Translation: Example – modify based on actual requirements.

Line 461:

Original: 对于其他模型，使用常规预测方法

Translation: Use standard prediction method for other models.

Line 465:

Original: 计算混淆矩阵

Translation: Compute confusion matrix.

Line 484:

Original: #############################新增模型评估######################################

Translation: New section: Model evaluation.

Line 485:

Original: 初始化结果存储

Translation: Initialize results storage.

Line 488:

Original: 对每个数据集进行分析

Translation: Analyze each dataset.

Line 490:

Original: cat("分析数据集:", dataset, "\n")

Translation: Print current dataset name.

Line 492:

Original: 获取该数据集的所有预处理方法

Translation: Retrieve all preprocessing methods for this dataset.

Line 495:

Original: 对每个预处理方法进行分析

Translation: Analyze each preprocessing method.

Line 497:

Original: cat(" 预处理方法:", premethod, "\n")

Translation: Print current preprocessing method.

Line 499:

Original: 获取该预处理方法下的所有算法

Translation: Retrieve all algorithms for current method.

Line 502:

Original: 如果至少有两个算法，进行两两比较

Translation: If more than two algorithms, perform pairwise comparisons.

Line 504:

Original: 提取所有算法的重抽样结果

Translation: Extract resampling results of all algorithms.

Line 513:

Original: 进行所有可能的两两比较

Translation: Perform all pairwise comparisons.

Line 520:

Original: 检查两个算法都有重抽样结果

Translation: Ensure both algorithms have resampling results.

Line 525:

Original: 确保长度相同且没有缺失值

Translation: Check for equal length and no missing values.

Line 529:

Original: 执行配对t检验

Translation: Conduct paired t-test.

Line 533:

Original: cat(" t检验错误:", e$message, "\n")

Translation: Print t-test error message.

Line 538:

Original: 计算效应量 (Cohen's d)

Translation: Calculate effect size (Cohen's d).

Line 543:

Original: 存储结果

Translation: Store results.

Line 560:

Original: cat(" 比较:", alg1, "vs", alg2, "- 完成\n")

Translation: Print comparison result message.

Line 569:

Original: 处理结果

Translation: Process result data.

Line 571:

Original: 将结果转换为数据框

Translation: Convert results to data frame.

Line 574:

Original: 应用多重比较校正

Translation: Apply multiple comparison correction.

Line 577:

Original: 标记显著性

Translation: Mark significance.

Line 582:

Original: 按数据集和预处理方法分组查看结果

Translation: Group results by dataset and preprocessing method.

Line 583:

Original: print("HCMM数据集结果:")

Translation: Print results for HCMM dataset.

Line 586:

Original: print("LCMM数据集结果:")

Translation: Print results for LCMM dataset.

Line 589:

Original: 保存结果为CSV文件

Translation: Save results as CSV file.

Line 592:

Original: 筛选显著结果

Translation: Filter significant results.

Line 594:

Original: print("显著结果:")

Translation: Print significant results.

Line 598:

Original: cat("没有找到任何可比较的结果\n")

Translation: No comparable results found.

Line 602:

Original: 2. Raw+pls/svm的模型里面，根据train的数据进行了pls-vip和svm-rfe的特征筛选，选前200个特征进一步再进行建模，然后想得到在test里面的模型预测结果。然后是根据新建立的分别有200个特征的pls和svm模型，分别在lcmm和hcmm里，想得到在test里的模型预测概率值用来画roc，然后想画之前那样的概率分布散点图。

Translation: Build PLS/SVM models using top 200 features from training data, predict probabilities on test set, and visualize ROC and probability distribution.

Line 1035:

Original: ########top200波数光谱图##########

Translation: Spectral plot of top 200 wavenumbers.

Line 1200:

Original: ##########################特征筛选#####################

Translation: Feature selection.

Line 1204:

Original: 提取波数-Mac里正常显示，所以2,8改成1,7就行

Translation: Extract wavenumbers – displays correctly on Mac, so change 2,8 to 1,7.

Line 1232:

Original: 筛选出MM类别的数据

Translation: Filter data with MM class.

Line 1233:

Original: 全

Translation: All

Line 1235:

Original: 去3

Translation: Remove spectra with absorbance > 3.

Line 1237:

Original: 创建基础图形（全部波数，只显示MM类别）

Translation: Create base plot (all wavenumbers, MM class only).

Line 1239:

Original: 只保留 Wavenumber 和 Class 列

Translation: Keep only Wavenumber and Class columns.

Line 1240:

Original: 左连接

Translation: Left join.

Line 1241:

Original: 从 M1_MM 中选择 Wavenumber 和 Absorbance

Translation: Select Wavenumber and Absorbance from M1_MM.

Line 1242:

Original: 根据 Wavenumber 连接

Translation: Join by Wavenumber.

Line 1244:

Original: 重新排列列顺序

Translation: Reorder columns.

Line 1252:

Original: 创建基础图形（全部波数，只显示MM类别）

Translation: Create base plot (all wavenumbers, MM class only).

Line 1254:

Original: 全部波数使用虚线

Translation: Use dashed lines for all wavenumbers.

Line 1276:

Original: 填充色（如果形状可填充）

Translation: Fill color (if shape allows).

Line 1277:

Original: SVM特征点，使用红色三角形

Translation: SVM feature points: use red triangles.

Line 1279:

Original: 全部波数使用虚线

Translation: Use dashed lines for all wavenumbers.

Line 1301:

Original: 填充色（如果形状可填充）

Translation: Fill color (if shape allows).

Line 1302:

Original: SVM特征点，使用红色三角形

Translation: SVM feature points: use red triangles.

Line 1312:

Original: 全部波数使用虚线

Translation: Use dashed lines for all wavenumbers.

Line 1326:

Original: 填充色（如果形状可填充）

Translation: Fill color (if shape allows).

Line 1327:

Original: SVM特征点，使用红色三角形

Translation: SVM feature points: use red triangles.

Line 1329:

Original: 全部波数使用虚线

Translation: Use dashed lines for all wavenumbers.

Line 1343:

Original: 填充色（如果形状可填充）

Translation: Fill color (if shape allows).

Line 1344:

Original: SVM特征点，使用红色三角形

Translation: SVM feature points: use red triangles.

Line 1351:

Original: #合起来

Translation: Combine all plots.

Line 1355:

Original: 合并数据

Translation: Merge data.

Line 1358:

Original: 创建组合图

Translation: Create composite plot.

Line 1379:

Original: 同时映射fill和shape

Translation: Map both fill and shape.

Line 1389:

Original: 合并数据

Translation: Merge data.

Line 1392:

Original: 创建组合图

Translation: Create composite plot.

Line 1413:

Original: 同时映射fill和shape

Translation: Map both fill and shape.
