# Supplementary figures and images for: Near-infrared spectroscopy combined with machine learning for plasma-based discriminant diagnosis of malignant mesothelioma: a retrospective study
Source: PeerJ. 2025 Dec 19;13:e20503. doi: 10.7717/peerj.20503 (PMC12721102; doi:10.7717/peerj.20503)

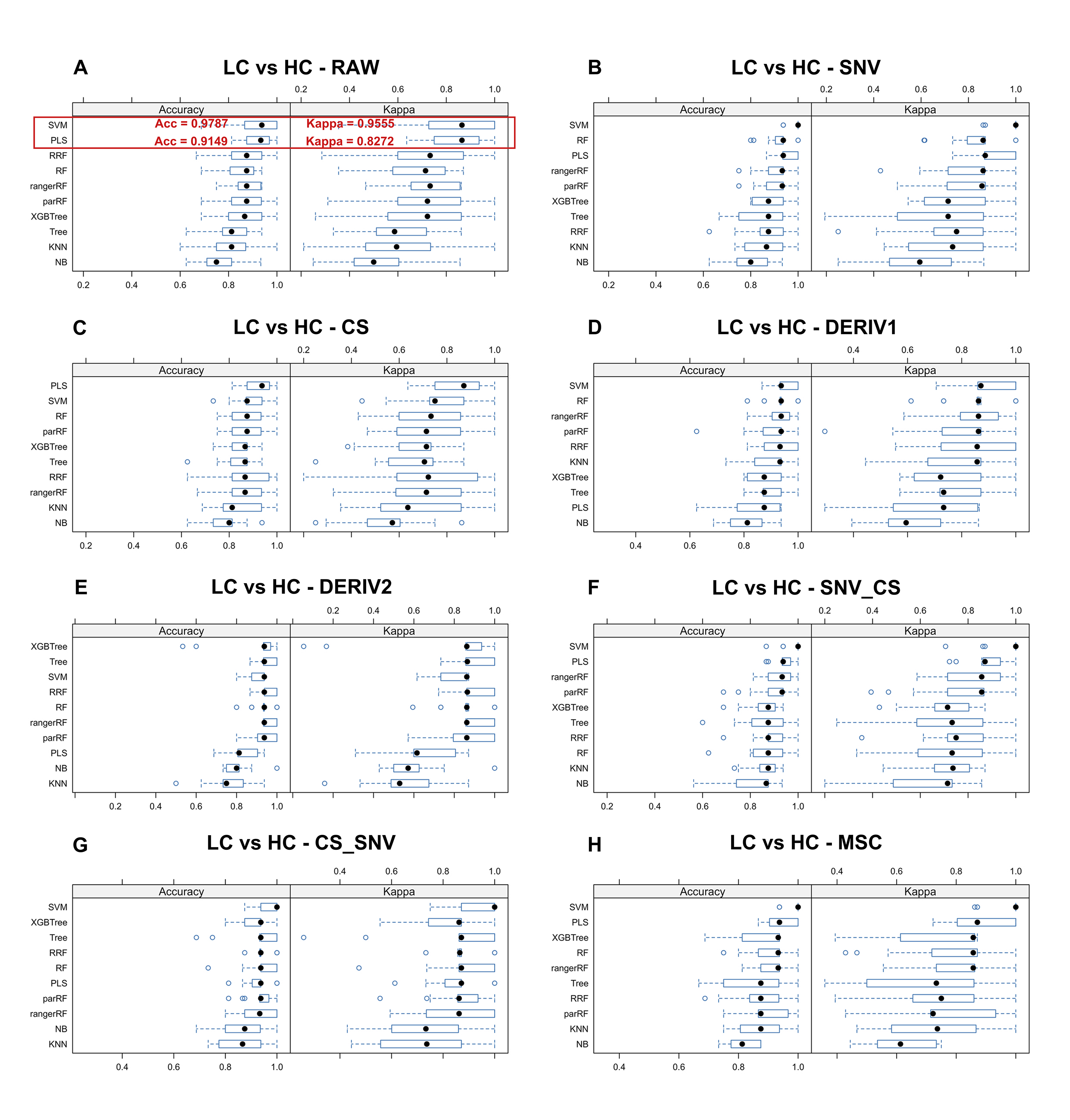

Supplement: Supplemental Information 4 — No samples were excluded based on this assessment. [file peerj-13-20503-s004.png]

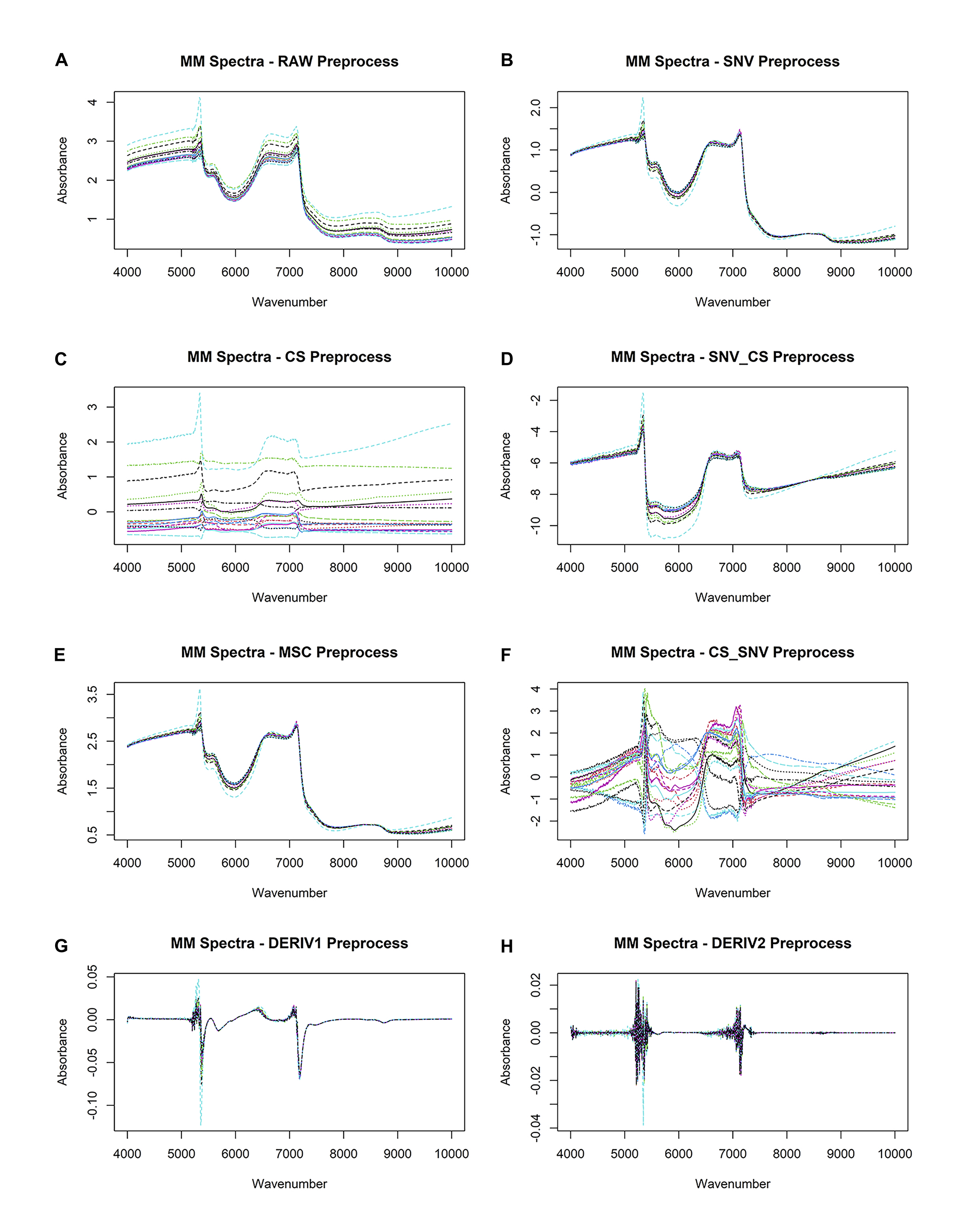

Supplement: Supplemental Information 5 [file peerj-13-20503-s005.png]
